# Epigenetic modification-related mechanisms of hepatocellular carcinoma resistance to immune checkpoint inhibition

**DOI:** 10.3389/fimmu.2022.1043667

**Published:** 2023-01-04

**Authors:** Shengwei Tao, Shuhang Liang, Taofei Zeng, Dalong Yin

**Affiliations:** ^1^ Department of Hepatobiliary Surgery, The First Affiliated Hospital of University of Science and Technology of China (USTC), Division of Life Sciences and Medicine, University of Science and Technology of China, Hefei, Anhui, China; ^2^ Department of Gastrointestinal Surgery, The First Affiliated Hospital of University of Science and Technology of China (USTC), Division of Life Sciences and Medicine, University of Science and Technology of China, Hefei, Anhui, China

**Keywords:** tumor immunotherapy, immune checkpoint inhibitors, hepatocellular carcinoma (HCC), epigenetic modification, immune resistance mechanisms

## Abstract

Hepatocellular carcinoma (HCC) constitutes most primary liver cancers and is one of the most lethal and life-threatening malignancies globally. Unfortunately, a substantial proportion of HCC patients are identified at an advanced stage that is unavailable for curative surgery. Thus, palliative therapies represented by multi-tyrosine kinase inhibitors (TKIs) sorafenib remained the front-line treatment over the past decades. Recently, the application of immune checkpoint inhibitors (ICIs), especially targeting the PD-1/PD-L1/CTLA-4 axis, has achieved an inspiring clinical breakthrough for treating unresectable solid tumors. However, many HCC patients with poor responses lead to limited benefits in clinical applications, which has quickly drawn researchers’ attention to the regulatory mechanisms of immune checkpoints in HCC immune evasion. Evasion of immune surveillance by cancer is attributed to intricate reprogramming modulation in the tumor microenvironment. Currently, more and more studies have found that epigenetic modifications, such as chromatin structure remodeling, DNA methylation, histone post-translational modifications, and non-coding RNA levels, may contribute significantly to remodeling the tumor microenvironment to avoid immune clearance, affecting the efficacy of immunotherapy for HCC. This review summarizes the rapidly emerging progress of epigenetic-related changes during HCC resistance to ICIs and discusses the mechanisms of underlying epigenetic therapies available for surmounting immune resistance. Finally, we summarize the clinical advances in combining epigenetic therapies with immunotherapy, aiming to promote the formation of immune combination therapy strategies.

## 1 Introduction

Primary liver cancer is the sixth most commonly diagnosed malignancy and the third leading reason for cancer mortality worldwide, accompanied by an extremely high number of new and fatal cases (1).

Hepatocellular carcinoma (HCC) constitutes 75%-85% of primary liver cancer as the commonest subtype. Potential Curative treatments, comprising surgical resection, liver transplantation, and local ablation, are amenable to early-stage HCC patients, which account for less than 20% of all HCC cases (2–4). However, most HCC patients are identified in the unresectable stage given its rapid progression, high malignancy, and inconspicuous early symptoms, resulting in palliative or symptomatic treatment, such as transarterial chemoembolisation (TACE) and systemic therapies (4, 5). Therefore, the identification of appropriate systemic therapy for advanced HCC has been an area of intense interest.

**Figure 1:**
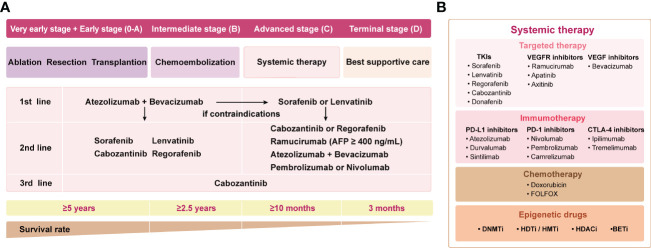
The landscape of systemic therapy for HCC treatment strategies. **(A)** The guidelines are sorted out and summarized by 2018 ASCO, 2018 AASLD, and 2020 EASL. The clinical stages of the patients are defined according to BCLC. **(B)** Main drugs for systemic therapy. TKI, Multi-tyrosine kinase inhibitor; VEGFR, vascular endothelial growth factor receptor; VEGF, vascular endothelial growth factor; ASCO, American Society of Clinical Oncology; AASLD, American Association for the Study of Liver Diseases; EASL, European Association for the Study of the Liver; BCLC, Barcelona Clinic Liver Cancer.

In recent years, with the advancement of precision cancer management, therapeutic strategies for HCC have emerged, including precision surgical resection, targeted molecular therapy based on different subtypes, and immunotherapy. Herein, the current landscape of systemic therapy for HCC treatment strategies is shown in **Figure 1A**. The application of multityrosine kinase inhibitor (TKIs) sorafenib as first-line therapy represented a milestone in systematic therapy (6). Despite the SHARPE trial and the ORIENTAL trial demonstrating improved median overall survival (OS) with sorafenib of 2.8 (10.7 vs 7.9)/2.3 (6.5 vs 4.2) months compared with placebo, respectively, anticancer efficacy remained suboptimal with a median survival of less than one year (6, 7). Notably, the therapeutic landscape has vigorously evolved after 2016 despite sorafenib remaining the pillar option for HCC patients during the past decade (5). Lenvatinib was approved for first-line treatment of advanced HCC due to its proven superior progression-free survival (PFS), and overall response rate (ORR) compared to sorafenib (8). In addition, regorafenib and cabozantinib were applied as second-line treatment since it was proven to prolong survival in progressed patients after suffering sorafenib treatment (9, 10). The biomarker-driven REACH-2 trial confirmed the efficacy of ramucirumab, especially in cases with baseline AFP >400ng/ml (11). Despite the promising outlook shown by these phase III studies, the OS benefits remain unsatisfactory.

During past decades, the application of T-cell immune checkpoint inhibitors (ICIs) such as cytotoxic T-lymphocyte-associated antigen 4 (CTLA-4) and programmed cell death protein 1 (PD-1)/programmed cell death-ligand 1 (PD-L1) monoclonal antibodies have completely opened the door to tumor immunotherapy (12–16). Currently, immunotherapy has achieved compelling efficacy in treating several solid and hematologic tumors, and promising clinical breakthroughs in the immune-based treatment of HCC are emerging (17–22). However, it is undeniable that a considerable number of patients experienced primary resistance to ICIs, despite ICIs offering prognostic improvement in certain groups (23). In addition, even patients who initially benefit may ultimately develop resistance, which means only a minority of patients have a long-lasting response to these treatments (19, 20, 24). Many hypotheses have been suggested to be responsible for primary nonresponse or acquired resistance to ICIs, focusing on the tumor-intrinsic factors or the tumor microenvironment (TME), such as lack of immunogenic epitopes, immunosuppressive cell populations, inflammatory phenotypes, and T-cell exhaustion (25–29). Therefore, discovering and identifying the causes of tumor immune escape and screening the best beneficiary population guided by predictive markers may lead to further breakthroughs in managing advanced HCC.

Nowadays, accumulating evidence has found that epigenetic modifications may act essentially in remodeling TME to avoid immune surveillance, affecting the efficacy of immunotherapy. This review mainly concentrates on the epigenetic changes in tumor cells, and the impact of TME reprogramming can be consulted in other reviews (30, 31). The history and advances in immunotherapy for HCC were introduced first, followed by a discussion of the mechanisms of HCC resistance to immunotherapy. Then, we summarize the rapidly emerging progress of epigenetic-related changes during HCC resistance to ICIs and discuss underlying epigenetic therapies available for surmounting immune resistance. Finally, this review summarizes the clinical advances in combining epigenetic therapies with immunotherapy, aiming to promote the formation of immune combination therapy strategies.

## 2 History and advances in Immunotherapy for HCC

**Figure 2:**
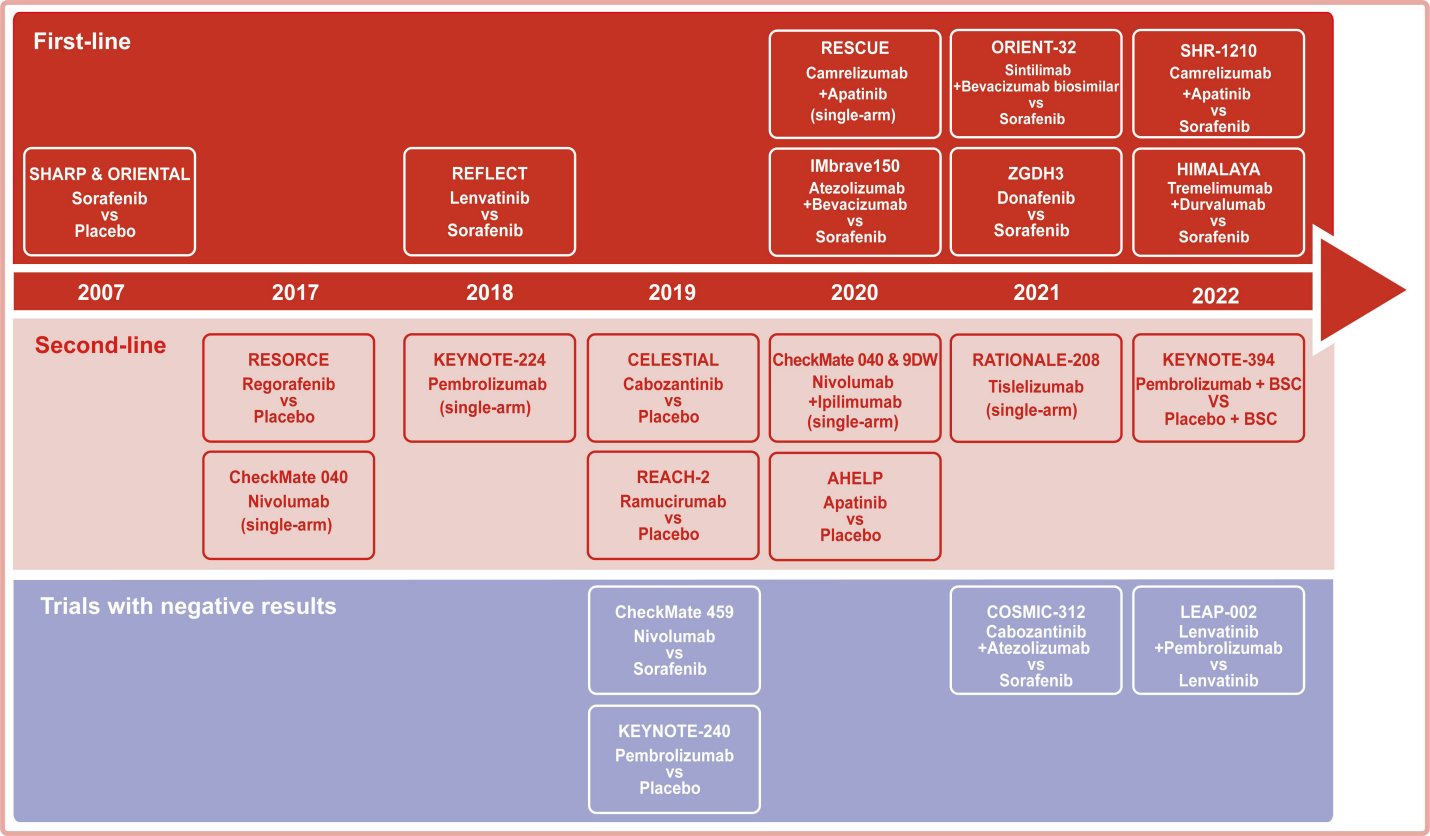
Representative clinical trials for the systemic treatment of HCC. Background color: red for trials with first-line settings, pink for trials with second-line settings, and purple for trials with negative results.

Systemic therapy based on TKIs and chemotherapy constituted the dominant treatment for advanced HCC before 2017. Notably, immunotherapies have been regarded as a breakthrough that has changed the treatment landscape for advanced HCC since 2017. Among immunotherapies, the success of ICIs therapy targeting PD-1, PD- L1, and CTLA-4 in solid and hematological tumors has shifted more attention to its potential in advanced HCC (**Table 1** and **Figure 2**).

**Table 1:** Representative clinical trials for the systemic treatment of HCC.

Publish Time	Identifier	Trial name	Agent	Target	Design	Phase	Endpoints	patients	ORR	mPFS in months	mOS in months
**Monotherapy for the treatment of advanced HCC**											
2017	NCT01658878	CheckMate 040	Nivolumab (single-arm)	PD-1	2 line	Phase I/II	Safety, tolerability	48 patients in dose-escalation phase	15%^a^	3.4 (1.6-6.9)	15.0
							ORR	214 patients in dose-expansion phase	20%^a^	4.0 (2.9-5.4)	15.6
2018	NCT02702414	KEYNOTE-224	Pembrolizumab (single-arm)	PD-1	2 line	Phase II	ORR	104	17%	4.9	12.9
2019/2022	NCT02576509	CheckMate 459	Nivolumab vs sorafenib	PD-1	1 line	Phase III	OS	743 (371 vs 372)	15% vs 7%	3.7 vs 3.8	16.4 vs 14.7
2019	NCT02702401	KEYNOTE-240	Pembrolizumab vs placebo	PD-1	2 line	Phase III	OS, PFS	413 (278 vs 135)	18.3% vs 14.4%	3.0 vs 2.8	13.9 vs 10.6
2022	NCT03062358	KEYNOTE-394	Pembrolizumab + BSC vs placebo + BSC	PD-1	2 line	Phase III	OS	453 (300 vs 153)	13.7% vs 1.3%	2.6 vs 2.3	14.6 vs 13.0
**Combination therapy for the treatment of advanced HCC**											
**Combinations of two ICIs**											
2020	NCT01658878	CheckMate 040	Nivolumab + ipilimumab	PD-1 + CTLA-4	2 line	Phase I/II	Safety, tolerability, ORR	148 (50 vs 49 vs 49)	32% vs 27% vs 29%^a^	NA	22.8 vs 12.5 vs 12.7
Ongoing	NCT04039607	CheckMate 9DW	Nivolumab + ipilimumab vs sorafenib or lenvatinib	PD-1 + CTLA-4 vs TKI	1 line	Phase III	OS				
2021	NCT02519348	Study22	Tremelimumab 300 + durvalumab vs tremelimumab vs durvalumab vs tremelimumab 75 + durvalumab	CTLA-4 + PD-L1 vs CTLA-4 vs PD-L1 vs CTLA-4 + PD-L1	1/2 line	Phase I/II	Safety	332 (75 vs 104 vs 69 vs 84)	24.0% vs 10.6% vs 7.2% vs 9.5%^a^	2.17 vs 2.07 vs 2.69 vs 1.87	18.7 vs 13.6 vs 15.1 vs 11.3
2022	NCT03298451	HIMALAYA	STRIDE (tremelimumab + durvalumab) vs durvalumab or sorafenib	CTLA-4 + PD-L1 vs PD-L1 or TKI	1 line	Phase III	OS	1171 (393 vs 389 vs 389)	20.1% vs 17.0% vs 5.1%^a^	3.78 vs 3.65 vs 4.07	16.43 vs 16.56 vs 13.77
**Combinations of one ICI and VEGF inhibitor**											
2020	NCT02715531	GO30140	GroupA: atezolizumab + bevacizumab (single-arm)	PD-L1 + VEGF	1 line	phase Ib	OS	104	36% (30-50)^a^	7.3 (5.4-9.9)	17.1 (13.8-NE)
			Group F: atezolizumab + bevacizumab vs atezolizumab monotherapy	PD-L1 + VEGF vs PD-L1			PFS	119 (60 vs 59)	20% vs 17%^a^	5.6 vs 3.4	NR vs NR
2020/2021	NCT03434379	IMbrave150	Atezolizumab + bevacizumab vs sorafenib	PD-L1 + VEGF vs TKI	1 line	Phase III	OS, PFS	501 (336 vs 165)	30% vs 11%^a^	6.9 vs 4.3	19.2 vs 13.4
2021	NCT03794440	ORIENT-32	Sintilimab + bevacizumab biosimilar ( IBI305) vs sorafenib	PD-L1 + VEGF vs TKI	1 line	Phase III	OS, PFS	571 (380 vs 191)	21% vs 4%^a^	4.6 vs 2.8	NR vs 10.4
**Combinations of one ICI and one mTKI**											
2020	NCT03006926	KEYNOTE-524	Lenvatinib + pembrolizumab (single-arm)	TKI + PD-1	1 line	phase Ib	safety, tolerability, ORR, DOR	104	46%^b^	9.3	22
2022	NCT03713593	LEAP-002	Lenvatinib + pembrolizumab vs lenvatinib	TKI + PD-1 vs TKI	1 line	Phase III	OS, PFS	794 (395 vs 399)	26.1% vs 17.5%^a^	8.2 vs 8.1	21.2 vs 19.0
2020	NCT03463876	Rescue	Camrelizumab + apatinib (first-line vs second-line)	PD-1 + VEGFR	1/2 line	Phase II	ORR	190 (70 vs 120)	34.3% vs 22.5%^a^	5.7 vs 5.5	immature
2022	NCT03764293	SHR-1210	Camrelizumab + apatinib vs sorafenib	PD-1 + VEGFR vs TKI	1 line	Phase III	OS, PFS	543 (272 vs 271)	25.4% vs 5.9% (P<0.0001)^a^	5.6 vs 3.7	22.1 vs 15.2
2021	NCT03755791	COSMIC-312	Cabozantinib + atezolizumab vs sorafenib	TKI + PD-L1 vs TKI	1 line	Phase III	OS, PFS	649 (432 vs 217 vs 188)	NA	6.8 vs 4.2	15.4 vs 15.5

^a^ according to Response Evaluation Criteria in Solid Tumors (RECIST) version 1.1; ^b^ according to modified RECIST.

ORR, overall response rate; OS, overall survival; PFS, median progression-free survival; VEGF, vascular endothelial growth factor; NA, not available; NR, not reached; NE, not estimable, NS, not significantly different; RECIST, Response Evaluation Criteria In Solid Tumors.

### 2.1 Monotherapy for the treatment of advanced HCC

Monotherapy employing antibodies targeting PD-1, PD-L, and CTLA-4 for the treatment of HCC, as outlined in **Figure 1B**. Well-known CheckMate 040 trial (NCT01658878) and KEYNOTE-224 trial (NCT02702414) were the first phase II ICIs trials to have proven promising results, confirming the significant efficacy of nivolumab and pembrolizumab in patients with advanced HCC (19, 20). CheckMate 040 was an open-label, non-comparative, phase I/II dose escalation and expansion trial, enrolling 262 patients with or without sorafenib exposure, demonstrating an ORR of 15% and 20% for nivolumab in the dose-expansion phase and dose-escalation phase, respectively (19). The median OS was 15.6 months in patients treated with nivolumab as second-line therapy, whereas the median OS was 28.6 months with nivolumab in the first-line setting without prior sorafenib therapy. Similarly, another phase II trial KEYNOTE-224, which enrolled 104 patients to evaluate the potential of pembrolizumab as second-line therapy, demonstrated an ORR of 17%, and a median OS of 12.9 months (20). Subsequently, the exciting results of these two trials led to accelerated approvals by the United States Food and Drug Administration (FDA) for nivolumab and pembrolizumab monotherapy as second-line therapy after sorafenib failure for progressive HCC.

Based on positive results from the uncontrolled single-arm trials, some randomized phase III trials were performed to validate the efficiency of monotherapy. CheckMate 459 (NCT02576509) is an open-label, randomized phase III trial that evaluated the efficacy of nivolumab and sorafenib as first-line treatment for advanced HCC in 743 patients (32, 33). The results showed that while patients treated with nivolumab showed a survival benefit of 1.7 months more than those treated with sorafenib, the trial did not meet the statistical significance of its predefined OS endpoint (median 16.4 months versus 14.7 months, HR=0.85; P=0.075). Furthermore, another randomized phase III trial KEYNOTE-240 (NCT02702401) compared the efficiency of pembrolizumab versus placebo as second-line therapy for 413 patients who progressed on previous sorafenib (34). Although the results showed significantly higher median OS and ORR in the pembrolizumab group than in the placebo group, the predefined OS and PFS statistical threshold were still not reached. In spite of demonstrated antitumor activity for nivolumab in the first-line setting and pembrolizumab in the second-line setting, the results of two phase III trials failed, which can be attributed to treatment with new drugs or ICIs after disease progression in the control group. Rewardingly, a recent phase III trial KEYNOTE-394 (NCT03062358) enrolled 453 Asian patients who failed in first-line treatment with sorafenib or oxaliplatin, confirming the remarkable benefits of second-line therapy with pembrolizumab in OS, PFS, and ORR (35). To date, other ICI monotherapies are currently being explored, and detailed results are presented in **Table 1**.

### 2.2 Combination therapy for the treatment of advanced HCC

The dilemma of ICIs monotherapy suggests that combining with other drugs to potentiate the efficacy of ICIs may be a promising direction. Several combination strategies are exhibiting outstanding results, including the combined application of two ICIs, one ICI plus vascular endothelial growth factor (VEGF) inhibitor, and one ICI plus one TKI. (**Table 1**).

#### 2.2.1 Combinations of two ICIs

The combination of nivolumab with ipilimumab was first evaluated by the CheckMate 040 trial, validating an ORR of 32% and a 2-year OS rate of 48% in child-Pugh class A patients previously treated with sorafenib (36). Positive data from this strategy led to accelerated FDA approval in 2020 for second-line treatment, and arm A was selected in an ongoing phase III study CheckMate 9DW (NCT04039607) for comparison with sorafenib or lenvatinib in first-line treatment.

Another regimen, termed STRIDE (Single Tremelimumab Regular Interval Durvalumab), has been shown to have clinical activity and manageable safety in phase I/II trial Study 22 (NCT02519348) (37). More recently, results from this regimen’s Phase III clinical trial HIMALAYA (NCT03298451) were also announced, demonstrating significantly improved ORR and three-year OS rates compared to sorafenib (38). In addition, durvalumab monotherapy was shown to be non-inferior to sorafenib in the first-line treatment. Hence, the STRIDE regimen was approved by FDA in October 2022 for the first-line treatment of unresectable HCC patients.

#### 2.2.2 Combinations of one ICI and VEGF inhibitor

The combination of atezolizumab and bevacizumab was explored in a phase Ib trial GO30140 (NCT02715531) that demonstrated an ORR of 36% and a median PFS of 7. 3 months (39). Given such results, a global, open-label, phase III randomized trial IMbrave150 (NCT03434379) was performed to validate the safety and efficiency of this strategy (40, 41). According to the updated study, median OS was prolonged by 5.8 months with atezolizumab plus bevacizumab compared to sorafenib, establishing its superior first-line status in the treatment of advanced HCC over sorafenib (41). This pioneering advance demonstrated the mechanistic synergy and efficacy of the combination of anti-vascular therapy and immunotherapy, inspiring subsequent clinical trials with ICIs in combination with TKIs or anti-VEGF monoclonal antibodies. For instance, a randomized, open-label, phase II/III trial ORIENT-32 (NCT03794440) enrolled 571 Chinese patients with unresectable HBV-associated HCC (42). The results showed that sintilimab plus bevacizumab biosimilar (IBI305) showed significant OS and PFS benefits in first-line therapy with a manageable safety profile compared to sorafenib.

#### 2.2.3 Combinations of one ICI and one TKI

A preclinical study revealed that the combination of lenvatinib and PD-1 inhibitors had a synergistic effect and increased efficacy (43). Therefore, a phase Ib clinical trial KEYNOTE-524 (NCT03006926) was performed to verify the safety and efficacy of lenvatinib plus pembrolizumab in patients with unresectable HCC (44). Based on the favorable safety and efficacy demonstrated by this regimen, a phase III trial LEAP-002 (NCT03713593) was subsequently conducted (45). Unexpectedly, although lenvatinib plus pembrolizumab was found to have elevated benefits in OS and PFS, it did not reach the predefined statistically significant difference. Nevertheless, the subgroup analysis of OS suggested that patients with portal vein invasion/extrahepatic metastases, HBV-related HCC, and APF >400ng/mL all benefited more from lenvatinib in combination with pembrolizumab. The regimen of Camrelizumab plus Apatinib has also been shown in Rescue (NCT03463876) and SHR-1210 (NCT03764293) to have positive efficacy and a manageable safety profile in first-line setting (46, 47). Another promising regimen, cabozantinib in combination with atezolizumab, was assessed in advanced renal cell carcinoma (48). However, the phase III trial COSMIC-312 (NCT0375579) of cabozantinib plus atezolizumab for the first-line setting of advanced HCC showed that the combination therapy significantly improved median PFS compared with sorafenib, but failed to improve median OS (49).

In the past few years, despite the failure of some clinical trials, several studies have shown exciting survival benefits, shifting to first-line treatment options (**Figure 2**). Meanwhile, the combination regimens also showed hidden dangers in tolerability and safety that need attention. Honestly, the efficiency of immunotherapy for HCC remains inadequate compared to other tumors with favorable responses. In fact, only a small percentage of patients with advanced HCC derive significant benefits from ICIs treatment. To address this need, efforts are required to focus on identifying molecular biomarkers that predict response to TKIs or ICIs and exploring novel drug mechanisms in combination with ICIs.

## 3 Mechanisms of HCC resistance to immunotherapy

**Figure 3:**
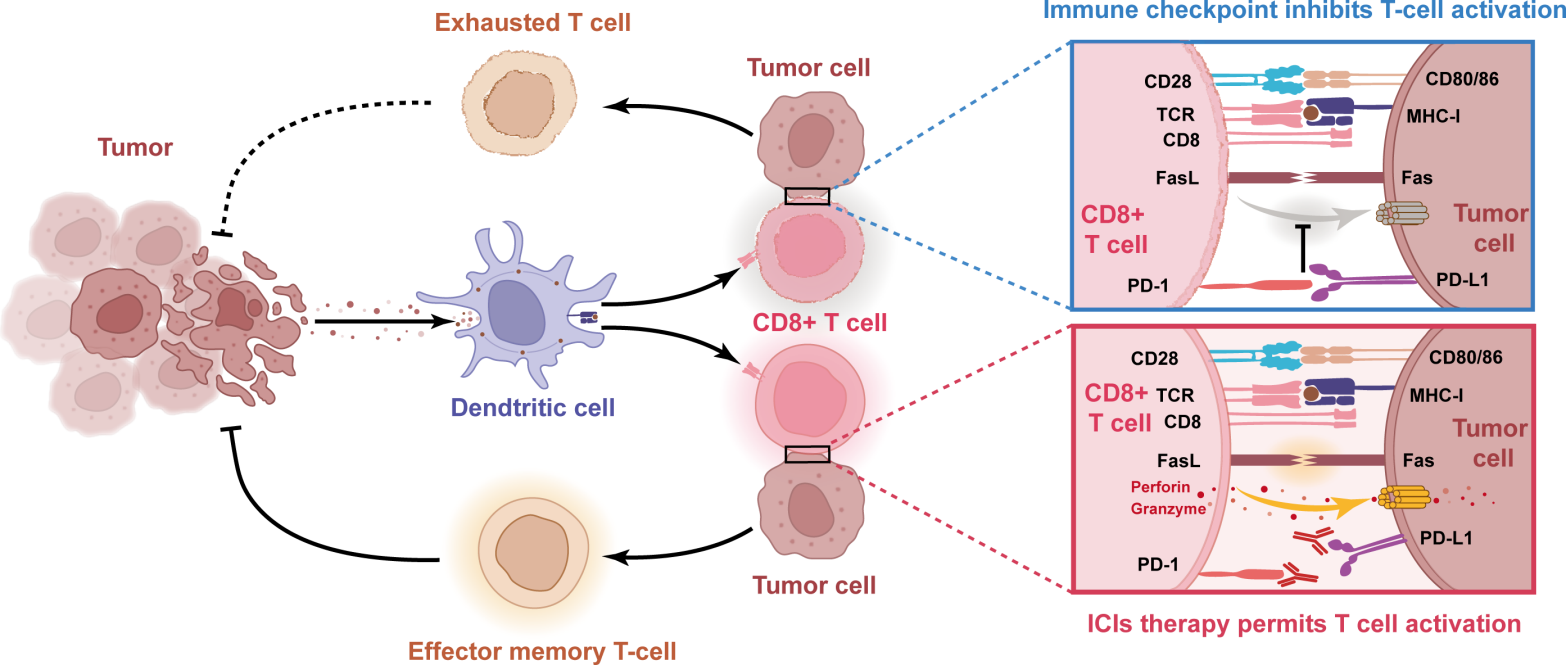
Schematic diagram of the molecular mechanism of impaired anti-tumor immunity caused by immune checkpoints and reactivation of T cells with PD-1/PD-L1 blocking antibody. When tumor cell PD-L1 binds to T cell PD-1, this interaction leads to T cell dysfunction and lack of anti-tumor activity. Thus, blocking the interaction between PD-1 and PD-L1 with anti-PD-1 or PD-L1 antibodies can reactivate T cells and release their anti-tumor activity.

**Figure 4:**
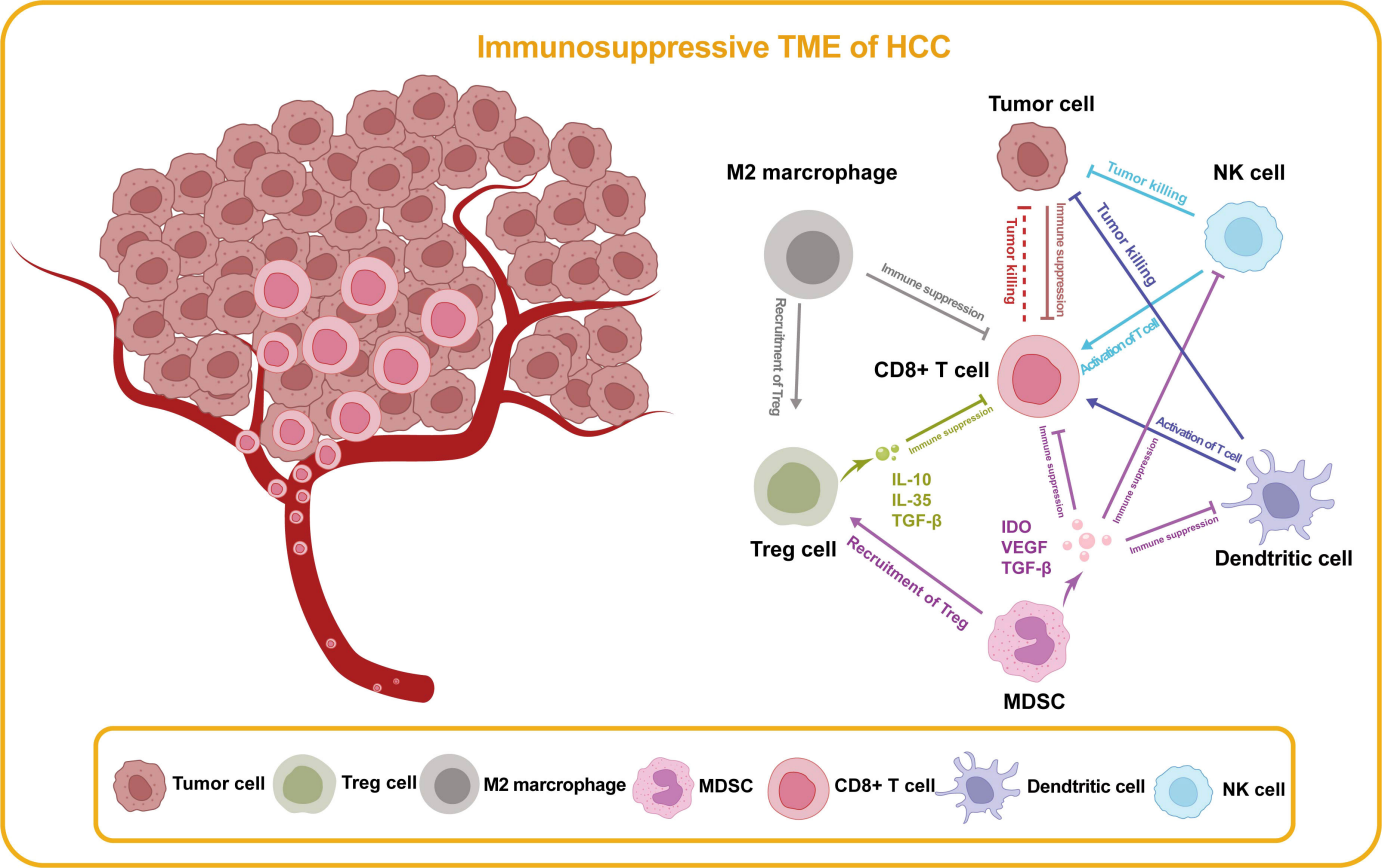
Immunosuppressive TME of HCC. In the TME of HCC, there are cell types that promote anti-tumor immunity and cell types that impede effective immune surveillance, which are illustrated in this figure. Treg cell, regulatory T cell; MDSC, myeloid-derived suppressor cell; NK, natural killer; IDO, indoleamine 2,3-dioxygenase; VEFG, vascular endothelial growth factor; TGF-β, transforming growth factor-β.

The hallmark of developing cancer is a dynamic immunoediting process that interacts with the immune system. This hypothesis explains the ability of immune cells to eliminate the tumor (immune surveillance) while shaping the tumor immunogenicity to produce an environment that contributes to tumor growth and progression (immune tolerance) (**Figures 3**, **4**). In the battle between the immune system and the tumor, there are three phases in sequence: immune elimination (early cancerous cells are recognized and eliminated by the immune system), immune equilibrium (sporadic tumor cells that survive immune elimination are not visible and cannot grow excessively), and immune escape (tumors gradually grow with distinct clinical features and establish an immunosuppressive microenvironment to evade killing by the immune system) (50, 51). For immunotherapy to be successful, three essential criteria need to be met: firstly, tumor antigen-specific T cell responses need to be activated; secondly, T cells need to infiltrate the TME; and finally, activated T cells need to trigger tumor cell killing mechanisms. Taken together, ICIs treatment failure can be caused by a defect in the above steps.

It is growingly recognized that epigenetic modifications occurring in tumor cells and immune cells within the TME represent the essential factors of cell growth, immune evasion, and drug resistance. Critical factors of primary response resistance to immune checkpoint blockade have been increasingly recognized, including tumor-intrinsic factors (tumor neoantigen burden and activation of oncogenic signaling pathways), TME (low tumor-infiltrating lymphocytes, exhausted CD8+ T cells, immunosuppressive cells and epigenetic silencing of chemokine), host immune components and microbiomes. Epigenetic modification-induced silencing of gene expression and loss of mutation-associated antigens, which impairs immunogenicity and immune recognition, have been implicated as one of the mechanisms of acquired drug resistance. For example, hypermethylation regulated by DNMTs and histone deacetylation regulated by HDACs contribute to the loss of function of tumor suppressor genes or immune presenting genes (e.g., MHC class-I expression), leading to tumor antigen presentation dysfunction and immune evasion (52). Moreover, activating mutations in CTNNB1 were correlated with a low response to ICIs monotherapy in advanced HCC patients (53). Despite multiple factors that might explain the therapeutic outcomes of ICIs, acquired resistance to HCC may be primarily attributable to the reprogramming of TME, which impedes the infiltration of lymphocytes (54–57).

## 4 Epigenetic modification-related mechanisms of HCC resistance to ICIs

**Figure 5:**
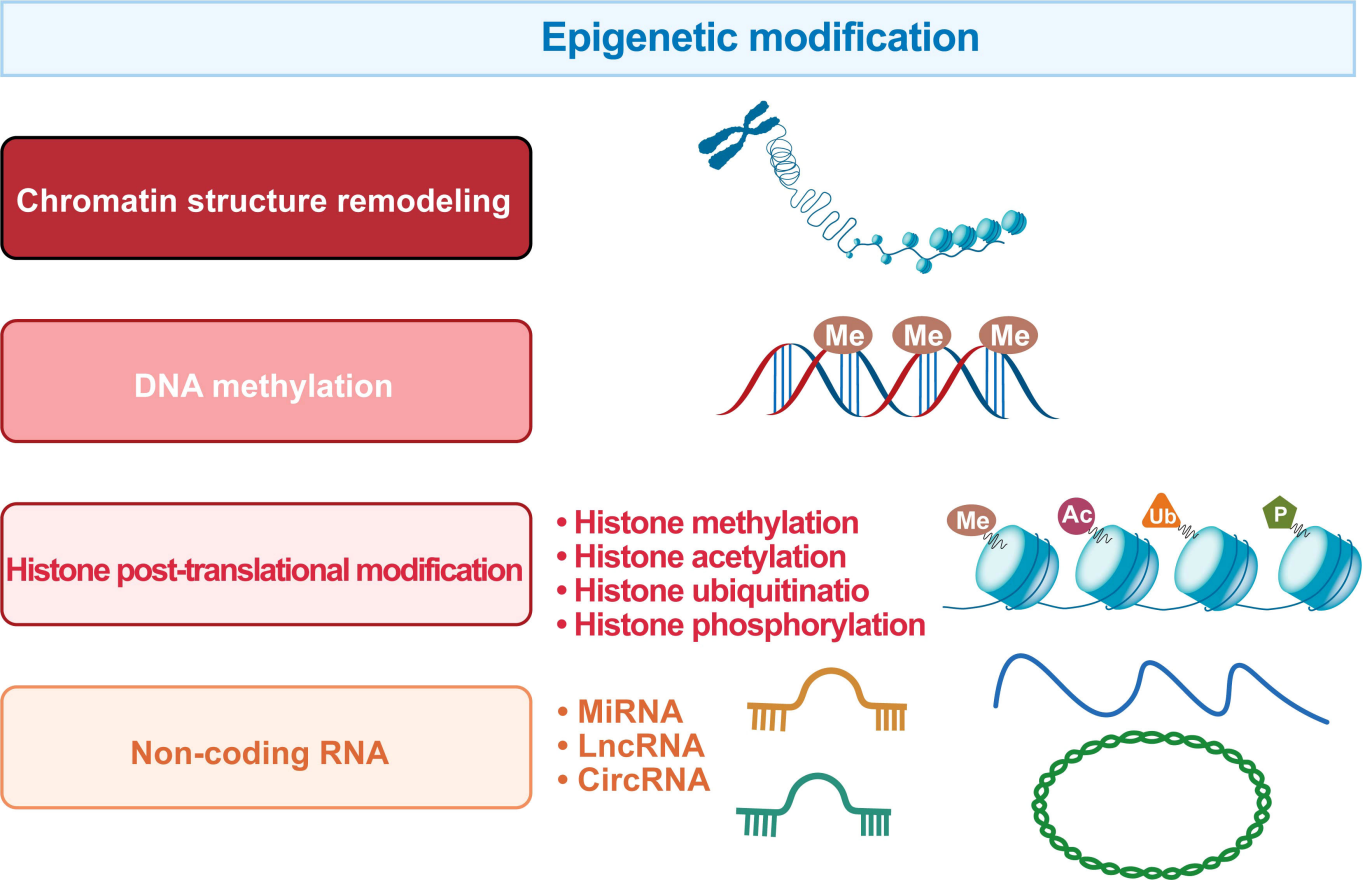
The regulatory systems involved in the epigenetic landscape of HCC. The epigenetic marks in HCC include chromatin structure remodeling, DNA methylation, histone post-translational modification and non-coding RNA.

Epigenetics is a mechanism that changes biological phenotypes without involving DNA sequence changes, and such changes can be passed on to offspring. Specifically, epigenetic modifications include chromatin structure remodeling, DNA methylation, histone post-translational modifications, and non-coding RNA levels (**Figure 5**).

### 4.1 Chromatin structure remodeling

Chromatin is a dynamic structure composed of DNA and histones, consisting of H2A, H2B, H3, H4, and DNA (58, 59). Chromatin conformation is essentially controlled by DNA modifications and histones subjected to post-translational modifications, leading to altered transcriptional activity (60, 61). The mating-type switch/sucrose non-fermenting (SWI/SNF) complex regulates chromatin accessibility in an adenosine triphosphate (ATP)-dependent nucleosome remodeling manner, which is the most intensively studied chromatin remodeling complex. Hence, its gene-encoding region is frequently mutated in tumors (62). The SWI/SNF complex is a macromolecular complex of 12-15 subunits, including a catalytic ATPase subunit, SWI/SNF related, matrix associated, actin-dependent regulator of chromatin, subfamily a, member 4 (SMARCA4), and several subunits, such as AT-rich interaction domain 1A (ARID1A) and ARID1B or polybromo 1 (PBRM1) and ARID2 (63). SWI/SNF subunit inactivating mutations, such as ARID1A, ARID1B, ARID2, PBRM1, and SMARCA4, are frequently detected in HCC (62).

ARID1A encodes one subunit of the SWI/SNF complex and regulates many processes requiring DNA access (such as transcription, DNA damage repair, and replication) by remodeling chromatin structure. ARID1A mutation was found to be associated with larger HCC and highly or moderately differentiated HCC (64). Moreover, He et al. found that ARID1A downregulation was associated with metastasis and poor prognosis in HCC, possibly due to the downregulation of E-cadherin (65). In addition, ARID1A mutations have also been reported to cause angiogenesis by upregulating angiopoietin-2 (Ang2) *via* H3K27ac modification (66). An *in vivo* experiment revealed that ARID1A knockdown could lead to mouse hepatocarcinogenesis, accompanied by macrophage and neutrophil infiltration and activation of STAT3 and NF-κB pathways (67). Notably, mutations in the SWI/SNF complex have been found to be involved in resistance to ICIs. ARID1A-deficient tumor-bearing mice exhibited increased mutational load as well as better lymphocytic tumor infiltration accompanied by increased PD- L1 expression in ovarian cancer. And ARID1A-deficient mice combined with anti-PD-L1 treatment were significantly more effective than mice with ARID1A wild-type ovarian tumors (68). Furthermore, inhibition of PBRM1, another SWI/SNF complex, was found to enhance immunotherapeutic response by increasing tumor immunogenicity (69). These results suggest that the aberrant SWI/SNF complex may engage in the TME of HCC and inhibition of the SWI/SNF complex may offer a combined effect with ICIs. More in-depth studies are needed to elucidate the mechanism of mutated SWI/SNF complexes shaping the TME.

### 4.2 DNA methylation

DNA methylation is achieved by adding a methyl group to cytosines at CpG sequences in gene-promoter regions by DNA methyl transferases (DNMT), resulting in gene silencing. The overall depletion of methylation at repetitive element regions that preserve genomic stability while exhibiting hypermethylation at promoter regions of tumor-suppressor genes is prevalent in cancer (70–73). In HCC, significantly elevated levels of DNA hypermethylation were observed in the promoters of genes associated with TP53, cAMP, serine proteases, and NADH regulation compared to normal tissues (74). Analysis of the cohort of human HCC versus normal tissues revealed the signature of tumor suppressor gene hypermethylation as detected by methylation-specific PCR (75). Moreover, genes regulated by methylation sites in non-tumor tissues of hepatitis cirrhosis potentially drive tumorigenesis and recurrence and carry significant prognostic value (76–78). During the progression of cirrhosis to advanced HCC, ascending DNA methylation can distinguish the normal liver from the diseased tissue (74, 79–81).

The mechanisms contributing to hepatocarcinogenesis and drug resistance due to dysregulated DNA methylation may be diversified. For example, the expression of DNMT and TET is altered during hepatocarcinogenesis (82, 83). DNMT3 is overexpressed in HCC and correlates with hypermethylation of promoters controlling 22 oncogenes (83, 84). High Dnmt3b expression in HCC is regulated by the IL-6/STAT3 signaling pathway, contributing to resistance to sorafenib and poor prognosis (85). DNA methylation in HCC may also alter genes involved in immune surveillance (86–89). In sorafenib-resistant HCC cells, overexpression of DNIMT1 is accompanied by PD-L1 expression, causing poor prognosis (90). PD-L1knockdown or drug interference reverses sorafenib resistance in HCC by restoring the expression of CDH1, an intercellular adhesion molecule that inhibits HCC metastasis, which is silenced by DNMT1 methylation (90). Subsequently, it was shown that the DNMT inhibitor 5-azacytidine combined with anti-PD-L1 therapy led to tumor regression accompanied by increased cytotoxic T-lymphocyte infiltration in mouse models compared to monotherapy (91). This mechanism is synergistic with immunotherapy by inducing upregulation of the T helper 1 (Th1)-type chemokines CXCL9 and CXCL10, causing effective T cell traffic to the TME. Furthermore, SGI-110 (Guadecitabine), a second-generation DNMT1 inhibitor, exhibited anti-carcinogenic and anti-angiogenic activity in a xenograft HepG2 model (75, 92). These studies suggest that inhibition of epigenetic modifiers may collaborate with ICIs by strengthening immunogenicity, remodeling effective T cell function, and modifying the immunosuppressive TME, making more exploration needed.

### 4.3 Histone post-translational modifications

**Figure 6:**
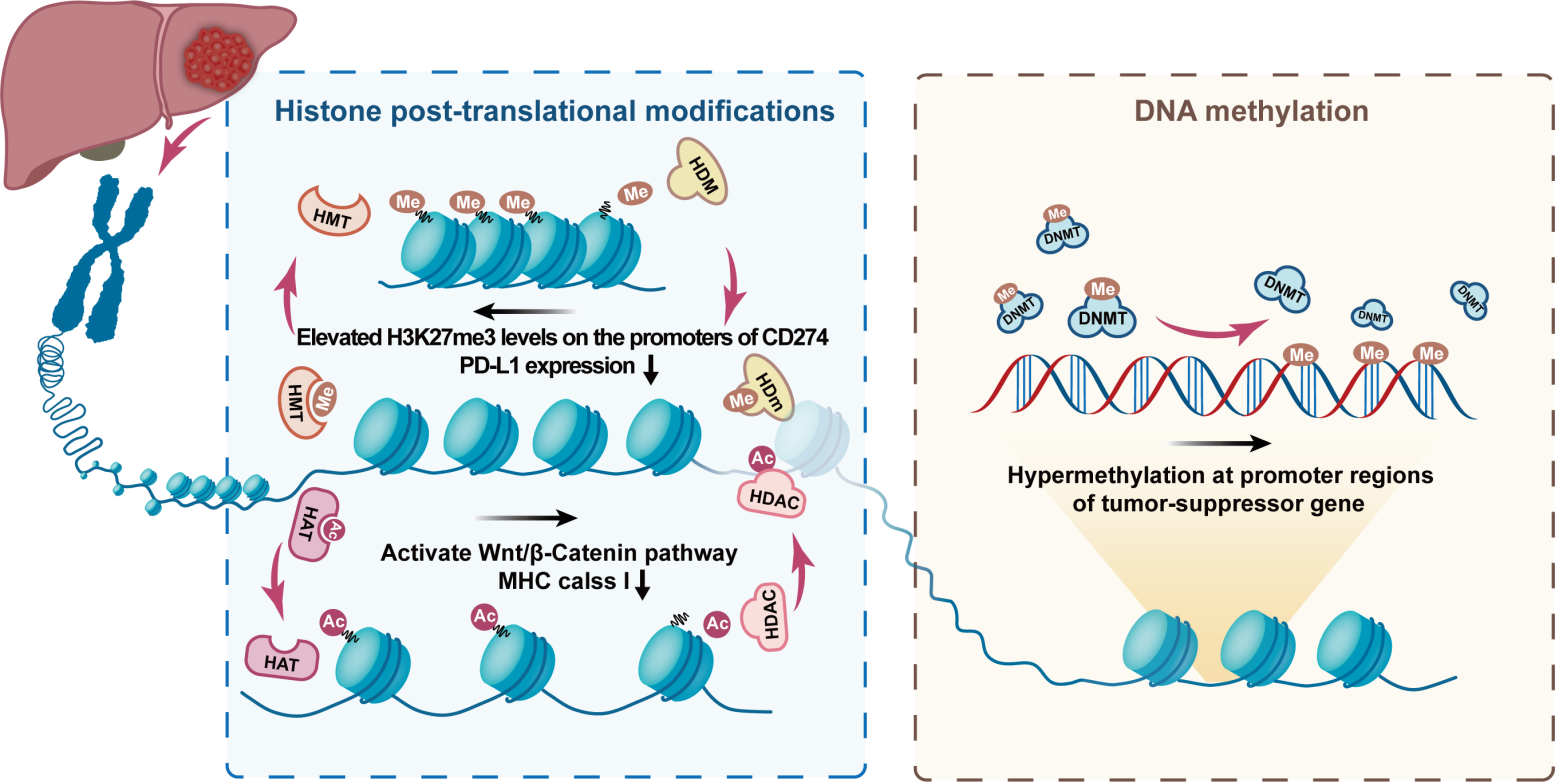
Typical histone post-translational modifications and DNA methylation mechanism. The figure shows the role of epigenetic regulation of chromatin by DNA methylation and histone post-translational modifications in the occurrence and development of HCC. The figure highlights the role of histone demethylase (HDM), histone methyltransferase (HMT), histone acetyltransferases (HAT), histone deacetylase (HDAC), and DNA methyltransferase (DNMT) in the formation of epigenetic characteristics of HCC. Ac, acetylation; Me, Methylation. The figure shows that EZH2 overexpression leads to elevated H3K27me3 levels on the promoters of CD274 and interferon regulatory factor 1 (IRF1), impeding PD-L1 expression. In, addition, the alteration caused by HDAC8 overexpression activates the Wnt/β-Catenin pathway, which in turn impairs antitumor immunity of antigen-specific T cells resulting in ICI resistance.

Histone modifications, including histone methylation, acetylation, ubiquitination, sumoylation, and phosphorylation, have been intensively studied in different cancers and are considered crucial factors for disease progression and immunotherapy resistance (93–96). The histone tails are modified to reversibly modulate chromatin compaction to promote or constrain accessibility to genes and further activate or silence gene transcription processes (97). Dysregulation of epigenetic modifiers of histones is recognized to exert an essential influence on hepatocarcinogenesis and immune escape (**Figure 6**).

#### 4.3.1 Histone methylation

The methylation of lysines on histones is mainly modulated by histone methyltransferases (HMTs) and histone demethylase (HDMs). In contrast to transcriptional activation due to histone acetylation, the effects of histone methylation are dictated by the location and number of residue methylation. For instance, the monomethylation of lysine 9 and 27 of histone H3 (H3K9me1 and H3K27me1) is correlated with an active chromatin state. In contrast, the trimethylations on lysine 9 and 27 of histone H3 (H3K9me3 and H3K27me3) are associated with a repressive chromatin (98).

Available evidence suggests that aberrant alterations in histone-modifying enzymes and anomalous histone modifications of genes involved in angiogenesis, cell cycle regulation, and cell adhesion are common in the progression of hepatocarcinogenesis. For example, H3K9me2 and H3K9me3 were overexpressed in HCC and correlated with a bad prognosis (99). Similarly, another study has also emphasized the critical role that high levels of H3K9me3 and its HMT SUV39H1 perform in the development and recurrence of HCC (100). The identical effect was also substantiated in other HMTs, such as SETDB1, G9a, and EHMT2 (101–105). Among the HMTs family, enhancer of zeste homolog 1 and 2 (EZH2), a catalytic subunit of the polycomb repressive complex 2 (PRC2), has recently captured much discussions. EZH2 is the crucial molecule of PRC2 and catalyses the trimethylation of H3 (H3K27me3) lysine residues to mediate the silencing of target genes (106, 107). A high association has been established between hyper-expressed EZH2 and HCC, indicating that EZH2 is tightly related to an aggressive phenotype and unfavorable prognosis (108, 109). Moreover, both EZH2 knockdown and drug blockade reduced the level of H3K27me3, resulting in reduced tumorigenesis (110).

The benefits of EZH2 inhibitors in combination with targeted therapy have begun to emerge. Kusakabe et al. observed overexpression of EZH2 and H3K27me3 in sorafenib-resistant cells, while the combination of sorafenib with an EZH2 inhibitor reversed sorafenib resistance resulting in a synergistic antitumor effect (111). Recently, it has been proposed that EZH2 can influence lymphocyte subpopulation differentiation and function to reshape the TME, and thus inhibition of EZH2 may be a novel strategy to improve anti-tumor immunity in certain cancers (112–114). Two H3K4me3-specific HMT, MLL1, and WDR5 have been proven to block immune escape and improve anti-PD-1 immunotherapy in pancreatic cancer (115, 116). Xiao et al. found that EZH2 potentiated H3K27me3 levels on the promoters of CD274 and interferon regulatory factor 1 (IRF1) to impede PD-L1 expression, suggesting that EZH2 inhibition combined with ICIs may provide accessible benefit (117). A study by Bugide et al. came to the identical conclusion, demonstrating that genetic or pharmacological inhibition of EZH2 induces re-expression of chemokine CXCL10 in HCC and therefore promotes migration and infiltration of NK cells into the tumor (118). The scope for clinical application of EZH2 inhibitors is being actively explored (119). Furthermore, given the outstanding inhibition of tumor growth demonstrated by EZH2 inhibitors in combination with anti-PD-1 therapy, Tazemetostat has been approved as the most widely studied EZH2 inhibitor as a first-line treatment option for epithelioid sarcoma (112, 114).

#### 4.3.2 Histone acetylation

The acetylation of lysines on histones is mainly controlled by histone acetyltransferases (HATs) and histone deacetylases (HDACs), which are responsible for mediating chromatin structural sparing and transcriptional activation of genes (120). Numerous studies have established a robust correlation between HCC and the dysregulation of HDAC. For example, Ler et al. revealed that HDAC1 and HDAC2 expression was upregulated in most HCC tissues, and HDAC1 expression is related to the degree of malignancy (121, 122). HDAC3 has been reported to be involved in DNA damage and repair processes, adjusting the balance between DNA damage and protumorigenic transcription (123, 124). hMOF, the histone acetyltransferase responsible for H4K16 acetylation, was found to have a dual effect of inhibiting growth and promoting vascular invasion in HCC, the exact mechanism of which remains unclear (125, 126).

Based on the currently available evidence, many efforts have focused on the function of HDAC in TME. Several studies have suggested that, in addition to altering the intrinsic phenotype of tumor cells, epigenetic therapies exhibit the potential to reverse primary or acquired resistance to ICIs. For example, Aberrant epigenetic modification by HDAC8 overexpression was demonstrated to play a crucial role in resistance to ICI (127). The alteration caused by HDAC8 overexpression activates the Wnt/β-Catenin pathway, which in turn impairs antitumor immunity of antigen-specific T cells resulting in ICI resistance (127, 128). Moreover, HDAC10 was found to recruit EZH2 enabling modification of the CXCL10 promoter region H3K27me3, producing CXCL10 transcriptional repression and therefore inhibiting NK cell migration and infiltration towards HCC (129). Therefore, inhibitors against HDACs and HATS are expected to be new targets for HCC management and require validation of effects verified in preclinical and clinical trials.

### 4.4 Non-coding RNA

Non-coding RNAs (ncRNAs) are not responsible for the translation into proteins and perform roles in regulating DNA methylation, histone modification, and gene silencing. Aberrations of ncRNAs have been reported extensively in HCC, and detailed information on these reports can be found in the following reviews (130–134). Given the immune-related focus of this review, representative ncRNAs have been selected for elaboration.

The expression of many microRNAs were closely associated with the degree of differentiation, and tumor metastasis of HCC, such as miR-497, miR-1246, and miR-378a-3p (135, 136). In addition, miR-378a-3p was also proved to directly regulate PD-L1 and STAT3 signaling to inhibit HCC, which may be a potential target in the future (137). Moreover, liver-derived exosome miR-92a-3p was identified as a potential biomarker for predicting HCC metastasis. Mechanistically, exosomal miR-92a-3p plays a key role in epithelial-mesenchymal transition (EMT) progression and promotion of metastasis through inhibition of PTEN and activation of the Akt / Snail signaling pathway (138). MiR-1 was found to be induced by NRF-2, promoting upregulation of PD-L1 expression and maintaining HCC resistance to sorafenib (139). MiR-200c inhibits PD-L1 expression by binding to the 3’ untranslated region (3’ UTR) of CD274 in HCC (140). Similarly, MIR-570 can also affect PD-L1 mRNA by binding its 3'UTR in HCC (141). The miR-144/451a cluster was also found to promote macrophage M1 polarisation and activity in a hepatocyte growth factor (HGF) and macrophage migration inhibitory factor (MIF)-dependent manner to inhibit HCC development (142). MiR-144/451a was also revealed to promote macrophage M1 polarisation in a specific cytokine-dependent manner to inhibit HCC development.

In terms of long non-coding RNAs (lncRNAs), KCNQ1 overlapping transcript 1 (KCNQ1OT1) was found to promote sorafenib resistance and PD-L1-mediated immune escape by sponging miR-506 and miR-146a-5p (143, 144). Cancer susceptibility candidate 11 (CASC11) was found to stabilize E2F transcription factor 1 (E2F1) mRNA by recruiting eukaryotic translation initiation factor 4A3 (EIF4A3), which in turn affects the activation of NF-KB signaling pathway and Pl3K/AKT/mTOR pathway, and further regulated PD-L1 expression (145). The above researches suggest that selected ncRNAs therapies combined with ICIs may be promising treatment candidates. It is encouraging that OTX-2002, the first mRNA therapeutic to downregulate MYC expression pre-transcriptionally through epigenetic regulation, has been approved for application in HCC patients. Currently, MYCHELANGELO I (NCT05497453) is evaluating the potential of OTX-2002 as a single agent or in combination with TKIs or ICIs.

## 5 The combination of epigenetic drugs with immunotherapies

**Figure 7:**
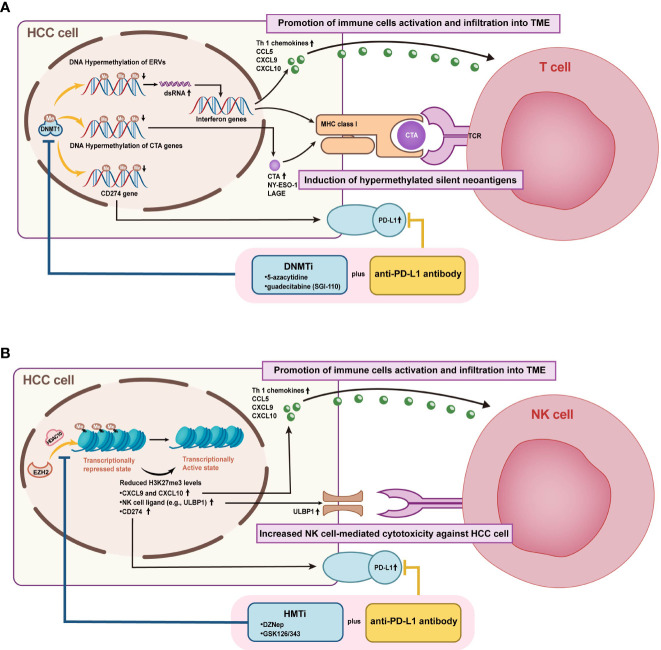
Mechanisms of combined epigenetic and immunotherapeutic strategies for HCC treatment. **(A)** Figure A illustrates the enhanced efficacy of DNMTi combined with ICIs by promoting immune cell activation and infiltration into TME and inducing hypermethylated silenced neoantigen expression. **(B)** Figure B demonstrates that HMTi combined with ICIs promotes immune cell activation and infiltration to TME and enhances NK cell-mediated HCC killing by upregulating the expression of chemokines, PD- L1, and NK cell ligand, which are inhibited by high histone methylation.

Unlike genetic mutations, epigenetic alterations are amenable to pharmacological interventions due to their flexible and variable interactions, rendering them a promising target for reversing ICIs resistance (146). Actually, epigenetic drugs not only exert a direct effect on tumors but also have the potential to remodel the suppressed TME and synergistically improve ICI efficacy (147–149). Currently, there is a wealth of research demonstrating the effectiveness of epigenetic modifiers drugs in many cancer models and pre-clinical applications (150, 151). In addition, some of these drugs have been intensively investigated in treating HCC. Therefore, it is reasonable to expect that coupling epigenetic drugs with ICIs may offer a desirable prospect for ICIs-resistant patients (**Figure 7**). Epigenetic modifications within TME were elaborated on in a previous review, and this review focuses on the epigenetic alterations occurring in HCC (30, 152). The current exploration of epigenetic drugs combined with ICIs in the clinic is summarized in **Table 2**.

**Table 2:** Epigenetics drugs in clinical trial.

Drugs	Phase	Identifier	Cancer type
**DNMTi**			
Guadecitabine (SGI-110) after sorafenib	Phase II	NCT01752933	HCC
Guadecitabine (SGI-110) + durvalumab	Phase Ib	NCT03257761	HCC
5-azacytidine (FT-2102)	Phase I/II	NCT03684811	HCC
5-azacytidine (FT-2102) + nivolumab	Phase I/II	NCT03684811	HCC
Epigallocatechin Gallate (EGCG)	Phase I	NCT02891538	Colon cancer
Epigallocatechin Gallate (EGCG)	Unknown	NCT01993966	Urothelial carcinoma
Decitabine	Phase I/II	NCT02316028	Unresectable liver metastases from colorectal cancer
Genistein	Phase I/II	NCT01985763	Colorectal cancer
Decitabine + genistein	Phase I/II	NCT02499861	Leukemias and solid tumors
Decitabine + genistein	Phase I/II	NCT01628471	Non Small Cell Lung Cancer
**HDACi**			
Trichostatin A	Phase I	NCT03838926	Hematologic malignancies
Resminostat (4SC-201) + sorafenib	Phase II	NCT00943449	HCC
Resminostat (YHI-1001) + sorafenib	Phase I/II	NCT02400788	HCC
Panobinostat (LBH589)	Phase I	NCT00873002	HCC
Sorafenib + panobinostat (LBH589)	Phase I	NCT00823290	HCC
Vorinostat (SAHA) + FOLFIRI	Phase I	NCT00537121	HCC
Vorinostat (SAHA) + sorafenib	Phase I	NCT01075113	HCC
Belinostat (PDX-101)	Phase I/II	NCT00321594	HCC
Tefinostat	Phase I/II	NCT02759601	HCC
Entinostat + nivolumab adenocarcinoma	Phase II	NCT03250273	Cholangiocarcinoma and pancreatic
Quisinostat	Phase II	NCT02948075	Ovarian cancer
Quisinostat	Phase I	NCT02728492	Non-small Cell Lung Cancer
Romidepsin + tenalisib	Phase I/II	NCT03770000	T-cell lymphoma
Romidepsin	Unknown	NCT02296398	Cutaneous T-cell lymphoma
Valproic Acid	Phase I	NCT01738815	Bladder cancer
**HMTi**			
GSK2816126	Phase I	NCT02082977	Neoplasms
CPI-1205	Phase I	NCT02395601	B-cell lymphoma
CPI-1205 + ipilimumab	Phase I	NCT03525795	Advanced solid tumors
Tazemetostat (Withdrawn)	Phase I	NCT03217253	Advanced solid tumors

### 5.1 DNA methyltransferase inhibitors (DNMTi)

DNMTi was shown to enable the demethylation of cancer-testis genes and repetitive sequences, leading to improved host immune surveillance by increasing immunogenicity (153). Currently, the inhibitors of DNMT include 5-azacytidine, decitabine, zebularine (ZEB), and guadecitabine (SGI-110). Some studies have also highlighted the association between DNA methylation and immune checkpoints or lymphocytic infiltration. In mouse models, the DNMT inhibitor 5-azacytidine reactivated the expression of TH1-type chemokines (CXCL9 and CXCL10), increased infiltration of cytotoxic T-lymphocyte infiltration, and caused tumor regression in combination with anti-PD-L1 (91). Besides resensitizing sorafenib-resistant cells, decitabine also brought PFS and OS benefits clinically (90, 154). SGI-110, a novel DNMTi, has already been demonstrated in a preclinical study to inhibit HCC growth and enhance the antitumor effects of sorafenib (75). Moreover, Liu et al. proposed that SGI-110 has the potential to combine immune checkpoint therapy by reactivating endogenous retroviral elements (ERVs) to stimulate immune response pathways (92). More clinical trials combining SGI-110 and immunotherapy are ongoing (NCT01752933, NCT03257761).

However, DNMTi does not seem to perform a favorable function necessarily. A previous study has reported that brain-expressed X-linked protein 1 (BEX1) methylation mediated by DNMT1 inhibited HCC stemness and tumorigenicity, while DNMT1i ZEB promoted self-renewal and invasiveness in the high cancer stem cell (CSC) score HCC group (155). They found that the promoter region of the BEX1 gene was highly methylated, and the activation of BEX expression by ZEB treatment aberrantly triggered the Wnt/β-catenin signaling pathway, causing the proliferation of tumor hepatocytes. Moreover, BEX overexpression was also found to lead to resistance to sorafenib, and knockdown treatment restored its sensitivity. Considering the differential effects of DNMTI, its exploration in different patient subtypes should be emphasized in subsequent studies to ensure a better survival benefit.

### 5.2 Histone demethylase/methyltransferase inhibitors (HDTi/HMTi)

The potential role of HMT and HDM inhibitors in managing HCC is also being actively pursued. For example, EZH2 negatively regulates PD-L1 in an IFN γ-dependent manner and might provide a promising target for combination immunotherapy (117). While previous studies demonstrate that EZH2 inhibitor GSK126 enhances transcription of NK cell ligands to promote natural killer cell-mediated HCC cell death, additional studies ought to be conducted to validate the benefit of EZH2 inhibitors in conjunction with ICIs (118). Furthermore, upregulation of another HMT, G9a, was defined to be significantly associated with HCC progression and aggressive clinicopathological features. Thus, inhibition of G9a may lead to novel approaches (104). Interestingly, the combination therapy of DNMTi and HMTi/HDTi also yielded therapeutic benefits. As an example, improved antitumor effects of ICIs combining 5-aza and 3-deazaneplanocin A were verified in a subcutaneous transplanted hepatoma cell model (91). CM-272, a dual inhibitor of G9a and DNMT1, exhibits anticancer efficacy *in vitro* and *in vivo* by restoring the differentiation phenotype of HCC cells (101). While CM-272 was proven to potentiate ICIs therapy in liver fibrosis and bladder cancer, the propensity in HCC remains to be established both at a fundamental and clinical phase (156, 157).

### 5.3 Histone deacetylase inhibitors (HDACi)

HDACi is clinically approved for treating hematologic malignancies that warrant continuous evaluation of its value in HCC. HDAC inhibitors such as TSA, panobinostat, valproic acid, and ITF2357 can inhibit HCC cells and may provide a combined effect with immunotherapy (158–161). TSA has been shown to improve the anticancer effects in combination with sorafenib (162). The pan-deacetylase inhibitor panobinostat demonstrated inhibitory effects by affecting the expression of angiogenic and epithelial-mesenchymal transition markers in the HCC model (163, 164). In addition, low expression of another HDAC, SIRT7, is proposed to induce global H3K18 acetylation and reactivate key metabolic and immune regulators, affecting tumorigenicity *in vitro* and *in vivo* (165). And it has been demonstrated that SIRT7 blockade stimulates PD-L1 expression, which provides a foundation for combining SIRT7 inhibitors with ICIs (166). Moreover, HDACi combined with DNMTi could provide stronger anti-proliferative effects compared to single agents in a xenograft HCC model (167).

Accumulating evidence has demonstrated that HDAC inhibition reprograms the TME to convert cold tumors into hot ones. HDAC inhibitor Belinostat was previously tested for its potential to treat advanced unresectable liver cancer in a multicenter phase I/II study (168). Later, a preclinical study demonstrated that belinostat has immune-mediated antitumor effects and may enhance the effectiveness of immunotherapy. In a subcutaneous Hepa129 mouse HCC model, Belinostat was observed to enhance anti-CTLA-4 antitumor activity by promoting early infiltration of M1 macrophages and suppressing regulatory T cells (169). Yang et al. observed that the inhibition of HDAC8 relieved T-cell hypo-infiltration by reversing H3K27 hypoacetylation occurring in the metabolic and immunomodulatory factor genome and activating T-cell transport chemokine expression in HCC (128). In a syngeneic and orthotopic C57BL/6 mouse Hepa1-6 hepatoma model, selective HDAC8 inhibitor PCI -34051 combined with anti-PD-L1 therapy improved tumor-infiltrating CD8+ T cells, eliciting an effective and up to 15-month tumor-free response to ICB. Comparably, HDAC2 inhibitors were also shown to block the transcription of immune checkpoint genes mediated by nuclear translocation of PD-L1, causing increased infiltration of CD8+ cytotoxic T cells in HCC (170). Inhibition of HDAC6 also specifically triggers TH17 cell activation and strengthens the anti-tumor immune response (171). Considering the prominent regulatory effects of HDAC in hepatocarcinogenesis and TME, it is conceivable that the combination of HDACi and immunotherapy will result in a better survival benefit.

### 5.4 Histone reader protein inhibitors (BETi)

Satisfactory results were also obtained for targeted epigenetic readers. The expression of acetylated H3 and H4 reader BRD4 is augmented in HCC, and the BRD4 inhibitor JQ-1 was proven to suppress HCC proliferation (172–174). Importantly, targeting BRD4 also showed enhanced efficacy of ICI in the experimental HCC model. BET bromodomain inhibitor molibresib combined with anti-PD-L1 shows enhanced anti-tumor effects by decreasing Monocytic-MDSC and increasing tumor-infiltrating lymphocytes (TILs) in fibrotic HCC mouse models (112).

Taken together, several therapeutic approaches targeting epigenetic mechanisms can modify tumor progression and response to treatment, supporting great promise in combining epigenetic strategies with ICIs. However, efforts must also be devoted to further understanding the complex epigenome and its regulation, discovering and exploiting novel epigenetic mechanisms, and assessing the effectiveness and safety of these approaches. The clinical application of epigenetic modulators is far from being realized, and subsequent implements for evaluating safer and more effective combined ICIs in larger populations warrant adequate attention. As the results of these trials are reported, distribution patterns based on biomarkers will likely provide the greatest benefit to patients.

## 6 Biomarkers of ICIs responses in HCC

Despite remarkable advances in etiological prevention, diagnostic techniques, and treatment strategies, the 5-year survival rate for all stages of HCC is still only 18%, prompting clinicians and scientists to scout for bio-predictive marker models for ICIs efficacy based on tumor and TME (118, 119). Different immunocompetent subtypes respond differently to ICIs treatment. Through in-depth characterization of the high-resolution HCC immune landscape, better prognostic enhancement strategies may be developed for specific immunocompetent subtypes. Therefore, identifying molecular biomarkers predicting response to TKIs or ICIs remains a valuable area of research to be actively explored.

Although previous studies have attributed positive immune responses to PD-L1 staining, tumor mutational burden (TMB), and microsatellite instability (MSI) in many tumors, it does not apply to HCC (175–178). A clinical study found no significant differences in OS and relapse-free survival (RFS) between PD-L1 high- and low-expressing subgroups in 2979 HCC patients. On the flip side, CheckMate017 and OAK also found that non-small-cell lung cancer patients with negative PD-L1 expression could benefit from immunotherapy (179, 180). Meanwhile, PD-L1 expression levels did not associate with immune response in HCC, according to the CheckMate040 and Keynote224 studies (19, 20, 181). ICIs response does not correlate with mutational burden in HCC, as revealed by next-generation sequencing (NGS) (53, 177). Analysis of RNA-seq data in the TCGA database also revealed consistent results (182). The incidence of MSI-high or mismatch repair defects (dMMR) in HCC is estimated to be low (~3%); patients with high microsatellite instability did not indicate a high response rate (183, 184). Nonetheless, two recent studies showed that higher intra-tumoral frequency of PD-1^high^ CD8^+^ T cells and CD38^+^ CD68^+^ macrophages were correlated with better response to ICIs detected by flow cytometry and multiplex IHC (185, 186). Currently, the prognostic relationship between PD-L1 expression on tumor cells and TILs remains controversial (187, 188). Therefore, the importance of spatial heterogeneity of the HCC TME in the evaluation of ICIs efficacy markers deserves further evaluation.

Recently, a clinical study showed that Wnt/β-Catenin pathway mutations frequently occur in HCC patients resistant to ICIs therapy (189). Mechanically, activation of Wnt/β-Catenin leads to ICIs resistance by impairing antigen-specific T cell-mediated antitumor immunity, which is demonstrated by constructing tail vein injections of a transposon-based vector expressing MYC; p53−/− (190, 191). There is much evidence that cytokine and immune cell infiltration in TME may influence the outcome of ICIs. For instance, serum CD137 concentration and M1 macrophage infiltration were potential predictors for HCC patients treated with immune-combined anti-vascular therapy. Moreover, several studies have also found that TGF β attenuates the ICIs by limiting TIL within the tumor (192, 193). In addition, the etiology of HCC may also be responsible for the heterogeneity of patient response to immunotherapy. For example, the HBV-associated HCC microenvironment is more immunosuppressive and exhaustive than the non-viral-associated HCC. The high enrichment of PD-1^high^ Tregs and PD-1^+^ CD8^+^ resident memory T cells in HBV-associated HCC implies an advantage of anti-PD-1therapy (194–196). Notably, a previous study found that the gut microbiome regulates chemokine-mediated immune cell accumulation *via* bile acids, affecting immune surveillance in HCC (197). Two recent clinical studies have demonstrated robust correlations between the gut microbiome and bile acids and the efficacy of ICIs therapy in HCC (198, 199).

Although several predictors have been identified, any single predictive biomarker has limitations and cannot effectively identify the beneficiary population. Using combined assays or building effective predictive models may improve predictive sensitivity and effectively capture the immune status of tumor patients. In the future, analyzing tumor and microenvironment characteristics through large samples and building multivariate models for immunotherapy efficacy prediction using machine learning and artificial intelligence will help develop a new paradigm for precision tumor therapy.

## 7 Conclusions

HCC is a malignancy that severely threatens human health, and most patients will progress to the advanced stage with a poor prognosis. Treatment for advanced HCC has been lacking effective means, and with the development of immunotherapy, HCC treatment is at the dawn of a new era. However, immune resistance and disappointingly low patient response rates are critical reasons plaguing efficacy improvement. As introduced by this review, epigenetic changes may be essential biomarkers for identifying immunotherapy responders, as well as promising targets for overcoming resistance to ICIs. The mechanisms that operate to drive drug resistance remain to be further elucidated.

Pretreatment of the microenvironment with epigenetic reagents before immunotherapy may help reprogram immune cells toward subtypes that are effective against cancer. The development of predictive biomarkers can help reveal the mechanism underlying ICIs resistance and the interaction mechanism within tumors and TME, which is crucial for individualized immunotherapy. Although our knowledge is still constrained, the current evidence indicates that epigenetic therapies exhibit sufficient potential. With the exploration of combination therapies (such as combining ICIs with TKIs or Epigenetic drugs) and immunotherapy guided by practical predictive markers to screen for optimal benefit populations, there may be further breakthroughs in managing advanced HCC.

## Author contributions

ST and DY contributed to designing, drafting, revising, and approving the final version of the manuscript. SL revised and reviewed the manuscripts. TZ provided help in software support and figure drawing. All authors contributed to the article and approved the submitted version.
